# Avapritinib and corticosteroids in advanced systemic mastocytosis with tumoral CMML and associated thrombocytopenia

**DOI:** 10.1093/omcr/omaf240

**Published:** 2025-12-26

**Authors:** Ouadii Abakarim, Ramzi Jeddi, Julien Rossignol, Olivier Hermine, Eric Delabesse, Pauline Condom, Camille Laurent, Teresa Botin

**Affiliations:** Cabinet d'Hematologie Agadir, Borj Alwahda, Amsernate, Agadir 80020, Morocco; Oncohematology Department, Castres-Mazamet Hospital, Castres 81108, France; French Reference Center for Mastocytosis (CEREMAST), Hôpital Necker, Assistance Publique-Hôpitaux de Paris, Paris 75015, France; Department of Adult Clinical Hematology, Hôpital Necker, Assistance Publique-Hôpitaux de Paris, 75015 Paris, France; French Reference Center for Mastocytosis (CEREMAST), Hôpital Necker, Assistance Publique-Hôpitaux de Paris, Paris 75015, France; Department of Adult Clinical Hematology, Hôpital Necker, Assistance Publique-Hôpitaux de Paris, 75015 Paris, France; Hematology Laboratory, Cancer University Institute of Toulouse Oncopole, Toulouse University Hospital, 31100 Toulouse, France; Medical biology Laboratory, Castres-Mazamet Hospital, Castres 81108, France; Pathology Department, Cancer University Institute of Toulouse Oncopole, Toulouse University Hospital, Toulouse 31100, France; Department of Haematology, University Hospital Galway, Galway H91 YR71 , Ireland

**Keywords:** systemic mastocytosis, chronic myelomonocytic leukemia, avapritinib, dexamethasone, thrombocytopenia

## Abstract

Systemic mastocytosis co-occurring with chronic myelomonocytic leukemia (SM-AHN) presents therapeutic challenges, especially in cases of cytopenia. We present a case of a 76-year-old woman with advanced SM-AHN and thrombocytopenia, who responded to reduced-dose avapritinib combined with dexamethasone. Initial treatment with midostaurin and azacitidine was discontinued due to hematological toxicity. Avapritinib (100 mg/day) initiated but reduced to 50 mg/day due to thrombocytopenia risk, with dexamethasone (20 mg) added for platelet support. Clinical improvement was observed within two weeks, with lymphadenopathy resolution, spleen and liver size reduction, platelet count normalization, and serum tryptase decrease. Avapritinib combined with dexamethasone offers a promising therapeutic strategy for SM-AHN, particularly in thrombocytopenic cases.

## Introduction

Systemic mastocytosis (SM) is a rare myeloid hematological neoplasm with a variable clinical presentation, classified into indolent or advanced forms. The indolent form is associated with a better quality of life, while the advanced form is linked to reduced overall survival (OS), with a median OS of 3.5 years [1]. Recent advances have improved understanding of its physiopathology, diagnosis, and treatment [2]. Diagnosis relies on identifying dense multifocal mast cell infiltrates (≥15 cells) in organ biopsies, atypical spindle-shaped cells, KIT gene mutations, CD2/CD25 expression, and elevated serum tryptase levels (>20 ng/ml) [3]. SM is often associated with AHN, notably chronic myelomonocytic leukemia (SM-AHN), with a median survival of 15 months [4]. Diagnosis of SM-AHN follows WHO 2022 criteria, with KIT D816V mutations found in bone marrow mast cells and sometimes circulating monocytes [5]. Additional mutations in ASXL1, SRSF2, and RUNX1, linked to poor prognosis, are also present.

Recent kinase inhibitors targeting the KIT D816V receptor have significantly improved outcomes in advanced mastocytosis. Midostaurin, a multi-kinase inhibitor, showed a 60% response rate and a median OS of 33.9 months. Avapritinib, a more selective inhibitor, achieved a 68% response rate and a median OS of 36 months [6, 7]. Both are now approved, with midostaurin used first-line in Europe. Avapritinib is recommended when platelet counts exceed 50 G/L due to bleeding risks, limiting its use in some patients with advanced disease, especially those with other associated hematologic neoplasms (AHN) affecting platelet formation.

Here we report the case of a 76-year-old woman with advanced SM-AHN with thrombocytopenia associated to lymphadenopathies who responded to a reduced dose of avapritinib combined with corticosteroid therapy.

## Case presentation

**Figure 1:**
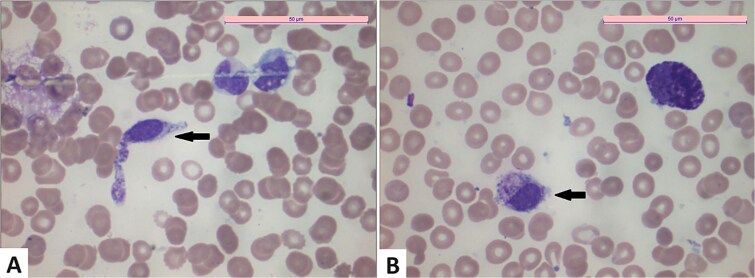
A) Spindle-shaped and degranulated mast cells, bone marrow, may Grünwald Giemsa staining, objective ×100; B) mast cells, including one degranulated mast cell, bone marrow, may Grünwald Giemsa staining, objective ×100.

A 76-year-old woman with no significant medical history was followed for CMML-1 since 2021. Clinical features included splenomegaly extending 5 cm below the costal margin and peripheral blood monocytosis at 2.75 G/L. Initial bone marrow aspiration revealed CMML with myelodysplastic features, a normal karyotype, and no excess of mast cells. Molecular analysis identified KRAS and SRSF2 mutations with intermediate variant allele fractions (VAF) and a low-level KIT D816V mutation (Fig. 1). No additional mutations were detected.

The patient was treated with hydroxycarbamide for myeloproliferative CMML, but the treatment was ineffective, leading to worsening anemia and thrombocytopenia.

A repeat bone marrow aspiration, performed to evaluate the aggravation of cytopenia, confirmed the CMML-1 diagnosis with 6% bone marrow blasts. Additional findings included 28% dystrophic mast cells without aggregates (Fig. 1), an increased KIT D816V mutation VAF of 2.9%, and a markedly elevated tryptase level of 389 ng/mL. These clinical, laboratory, and molecular features confirmed the diagnosis of SM-AHN.

**Figure 2:**
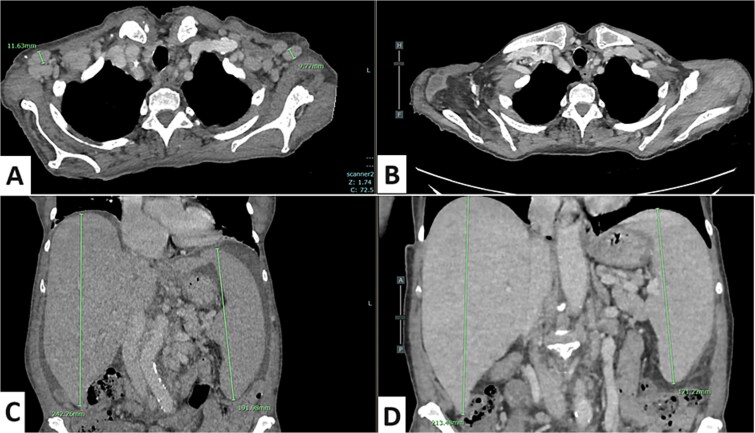
A) Homogenous bilateral axillary adenomegaly (minor axis between 10 and 14 mm); B) 3 months after treatment: complete regression of axillary adenomegaly; C) hepatomegaly (24 cm) and splenomegaly (19 cm) with ascites; D) hepatomegaly (21 cm) and splenomegaly (17 cm) and disappearance of ascitis after 3 months of treatment.

We treated her with midostaurin to reduce mast cell burden followed by azacitidine for the CMML were introduced but were suspended at 3 months because of hematological toxicity particularly with a severe thrombocytopenia. An overt tumor syndrome developed 3 months later, as assessed by CT (Fig. 2A, 2B), with massive hepato-splenomegaly and diffuse lymph nodes. A lymph node biopsy showed the existence of advanced extramedullary hematopoiesis secondary to advanced SM with an infiltration of mast cell aggregate, KIT and tryptase positive by immunohistochemistry analysis. Bone marrow aspiration showed abnormal mast cells (<10%) ruling out the diagnosis of mast cell leukemia.

Following discussion at a national multidisciplinary concertation meeting of the CEREMAST, avapritinib (100 mg/day) was started, with rapid dose reduction to 50 mg/day due to hematological toxicity, particularly thrombocytopenia and its risk of bleeding. In order to increase platelet count, weekly dexamethasone was added at an initial dose of 20 mg. The dose was tapered from 20 mg to 10 mg after three months, and then to 4 mg after the fourth month, which was maintained as a long-term weekly dose.

No non-hematological side effects were observed with either avapritinib or dexamethasone during the treatment course. The combination was well tolerated apart from the initial thrombocytopenia requiring dose adjustment.

**Figure 3:**
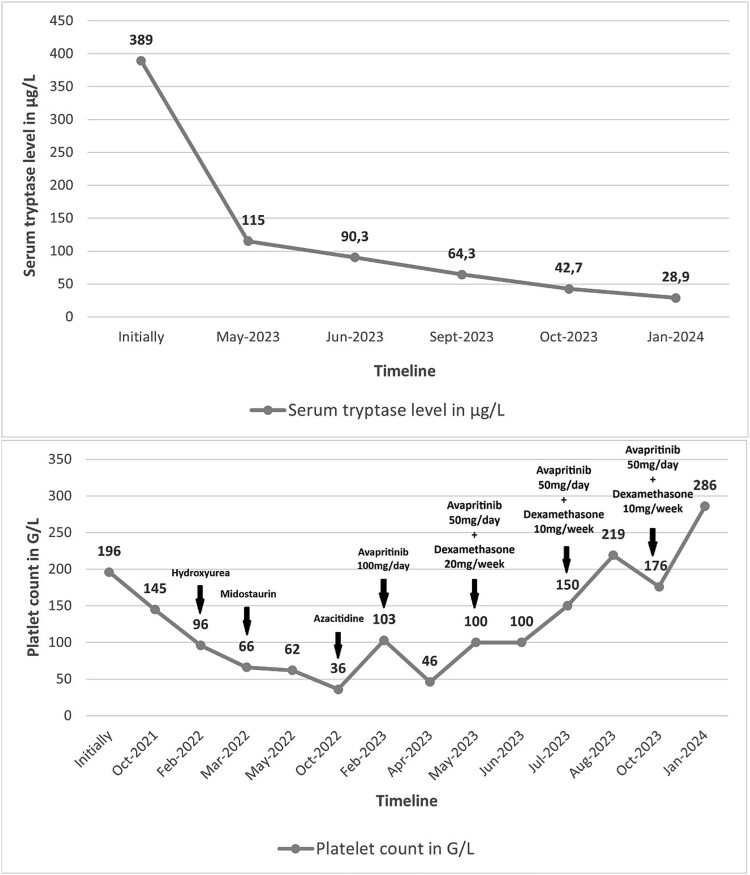
Evolution of serum tryptase level (A) and platelet count (B) according to timeline and treatment.

Evolution was marked by clinical improvement observed within two weeks of treatment initiation, with complete disappearance of the lymph nodes (Fig. 2C, 2D), significant reduction of spleen and liver size, normalization of platelet count, and a decrease in serum tryptase to 42 μg/L at the last dose (Fig. 3).

At present, the patient is on avapritinib 50 mg/day combined with dexamethasone 4 mg, maintained at 4 mg per week after a gradual reduction over 4 months.

## Discussion

SM-AHN is a distinct entity characterized by the coexistence of mast cell infiltration and concurrent hematologic malignancy. In this case, the patient had SM associated with CMML, a rare and challenging combination. Diagnosing SM-AHN requires a comprehensive approach, integrating clinical findings, bone marrow biopsy, cytological examination, molecular studies, serum tryptase levels, and imaging. The 2022 International Consensus Classification (ICC) and WHO 2022 (5th edition) classifications serve as critical tools for accurate classification and staging of this disease [3, 5, 8].

Therapeutic management of SM-AHN is complex, particularly to correct cytopenia particularly when associated with cytopenia. In such cases, it is vital to determine whether symptoms and cytopenias arise predominantly from the SM or the AHN component. Indicators such as elevated serum tryptase, significant mast cell infiltration, and a high VAF of the KIT D816V mutation may suggest prioritizing treatment of the SM component [1, 4].

Midostaurin, a multi-kinase inhibitor, is often the first-line treatment in advanced SM due to its efficacy in reducing mast cell burden and improving cytopenias in approximately 60% of cases. Moreover, midostaurin may also decrease monocyte counts in some patients with SM-AHN [9]. However, in cases of inadequate response or intolerance, avapritinib, a highly selective KIT D816V inhibitor, offers a potent alternative. Despite its efficacy, avapritinib carries a significant bleeding risk, particularly in patients with platelet counts below 50 G/L [6, 7].

In our case, the use of low-dose avapritinib combined with dexamethasone resulted in clinical improvement. Dexamethasone may enhance platelet counts, allowing safer initiation of avapritinib at a reduced dose, which can be titrated based on efficacy and tolerability. This combination therapy has shown promise in reducing mast cell and monocyte infiltration in bone marrow and peripheral tissues, including lymph nodes, as observed in prior studies [7, 10].

The presented case aligns with prior reports where avapritinib demonstrated efficacy in advanced SM, particularly when midostaurin was insufficient [6–10]. However, the concurrent use of corticosteroids, such as dexamethasone, has not been extensively documented, highlighting the novelty and potential of this approach in patients with severe thrombocytopenia.

This case emphasizes the importance of tailoring treatment in SM-AHN based on the underlying disease dynamics. For patients with advanced SM-AHN and thrombocytopenia, initiating treatment with reduced-dose avapritinib combined with dexamethasone may offer a safer and more effective strategy. Further studies are necessary to validate this approach and explore its long-term efficacy and safety.

## Conclusion

This case of SM associated with CMML and cytopenia showed a positive response to reduced-dose avapritinib combined with dexamethasone. These findings suggest that dexamethasone may be considered at the initiation of avapritinib treatment, particularly in patients with thrombocytopenia. However, additional studies and further evidence are required to validate this treatment strategy.
